# Solid or Liquid Food—The Intention to Eat Different Foods under Negative Emotions

**DOI:** 10.3390/foods11091180

**Published:** 2022-04-19

**Authors:** Chenjing Wu, Chuangbing Huang, Hongyan Zhu, Yuanlin Yu, Caiyun Zhang, Wei Zhang, Xianyou He

**Affiliations:** 1 Center for Studies of Psychological Application, Guangdong Key Laboratory of Mental Health and Cognitive Science, Key Laboratory of Brain, Cognition and Education Sciences, Ministry of Education, School of Psychology, South China Normal University, Guangzhou 510631, China; wuchenjing@m.scnu.edu.cn (C.W.); 2020023405@m.scnu.edu.cn (C.H.); 2020023463@m.scnu.edu.cn (H.Z.); 20202921062@m.scnu.edu.cn (Y.Y.); cheungwai@m.scnu.edu.cn (W.Z.); 2 School of Psychology, Northwest Normal University, 967 Anning Dong Lu, Lanzhou 730071, China; chengbao1026@163.com

**Keywords:** emotional eater, liquid food, negative emotion, solid food, the eating intention

## Abstract

Food can relieve an individual’s emotions, especially for emotional eaters. For instance, chewing alleviates negative emotions. Solid and liquid foods comprise a huge part of our daily lives, and the chewiness of solid foods is always high. Here, we explored whether people, especially emotional eaters, have higher eating intentions to eat highly chewy foods while experiencing negative emotions by comparing their eating intentions toward solid and liquid foods. To this end, we conducted a survey of 147 participants using a questionnaire (Experiment 1) to understand their eating intention toward five types of food (purple potato, maize, black soya bean, mango, and soybean; each food group contained a solid food and a liquid food) while experiencing negative emotions. The results showed that individuals exhibited higher eating intention toward solid food compared with liquid food while experiencing negative emotions. In Experiment 2, we selected 85 and 65 high-emotional and low-emotional eaters, respectively, and further explored their preference for solid foods. The results showed that individuals with high levels of emotional eating exhibited higher intentions toward solid food while experiencing negative emotions compared with those with low levels of emotional eating. In conclusion, this study proved that individuals’ higher eating intentions toward highly chewable food were pronounced among individuals with high levels of emotional eating under negative emotion conditions.

## 1. Introduction

For most individuals, eating is an extremely effective way to cope with negative emotions. Previous studies have shown that eating behaviors quickly relieve people’s negative emotions, such as sadness, anxiety, boredom, and pressure, which are subsequently replaced with positive emotions [1,2,3,4,5,6,7,8,9]. Generally, the effect of emotions on eating depends on the amount and type of food eaten [10,11,12]. With regard to the amount of food eaten, previous studies have suggested that people consume more food when experiencing both positive and negative emotions compared with when they do not experience any emotional swings [11,13,14]. Notably, obese individuals often exhibit increased food intake when experiencing negative emotions, and restrictive dieters, i.e., chronic dieters, are thus especially more likely to engage in eating upon experiencing negative emotions [15,16,17]. To date, however, the effect of negative emotions on the quantity of food intake remains unclear. Some researchers have found that negative emotions elicited excessive food intake, while others have reported the opposite trend [18]. In conclusion, the quantity of food intake is more or less affected by negative emotions [19]. The effect of emotions on the type of food eaten has also been documented [20,21,22,23,24]. For example, studies have shown that individuals in a positive emotional state tend to consume healthy food, while negative emotions have been associated with a tendency to consume junk food [25,26,27].

Participants in negative emotional states tend to choose high-fat, high-calorie foods [28]. Furthermore, studies have found a positive association between people’s experience of negative events with the consumption of high-sugar, high-fat, and high-energy foods [29,30,31,32,33]. In addition to foods high in sugar and fat, emotions also influence carbohydrate intake [34,35]. For example, obese women and those craving carbohydrates were found to ingest significantly more carbohydrates after dysphoric mood induction [29]. Individuals’ preferences and eating intentions for different types of food in emotional states are mainly influenced by the positive effects that the foods have on the alleviation of negative emotions [3,6]. For example, researchers have found that carbohydrate ingestion increases the secretion of certain neurotransmitters by the brain, a phenomenon that has been associated with mood improvement [36,37].

Previous studies on emotional relief have demonstrated that chewing behavior influences an individual’s emotions. Chewing is a rhythmic, complex process accompanied by neurological reflexes that involves various activities, such as facial communication, as well as blood circulation and secretion. A previous study showed that chewing behavior decreased anxiety emotions in rats [38]. In addition, it reduced mastication-activated monoamine transmitters in the hippocampal region to some extent and decreased negative emotions. The inhibition of neurotransmitters in critical areas was associated with an increased tendency toward anxious emotions [39]. Moreover, chewing was found to reduce corticosterone secretion in mice [40,41]. A previous study demonstrated that chewing gum had a significant impact on the alleviation of negative emotions [42], and Smith [43] found that chewing gum improved individual performance upon exposure to noisy conditions (a method of examining the effects of stress) [43]. The results showed that chewing gum resulted in higher alertness and a more upbeat emotional state. Heart rates were higher when gum was chewed, indicating that it had refreshing effects. Furthermore, Sasaki-Otomaru et al. found that subjects who chewed gum had lower anxiety than their non-chewing counterparts [44]. Overall, these results have demonstrated that chewing is significantly associated with emotion regulation. Since solid foods are more chewable than liquid ones, the act of chewing can be emotionally soothing. To date, however, it remains unknown whether people experiencing negative emotions also have different preferences for different chewable foods, which necessitates further research exploration. Emotional eating is a type of eating in response to a range of negative emotions, such as anxiety, depression, anger, and loneliness. Previous research has shown that emotional eating has a negative effect on emotional regulation [13,45]. It is commonly believed that when confronted with emotional events, emotional eaters cannot use effective emotional regulation strategies and only eat to diminish negative emotions [46,47,48]. That is, emotional eaters have a greater preference for using the act of eating to relieve their emotions.

### The Present Research

According to the research summarized above, we found that the effect of emotional eating, a trait closely associated with eating behavior, on the intention to eat different chewable foods in response to negative emotions remains unknown.

In the present study, we explored the eating intentions associated with different chewable foods in the context of negative emotions. Since emotional eaters tend to eat in order to alleviate their emotional states, which may lead to a higher eating intention for highly chewy foods, we evaluated whether this eating intention for different chewable foods changes with emotional eating, with the aim of providing some support for the marketing and consumption of food. Solid and liquid foods are the two most common categories of daily foods. Generally, solid foods are more chewable than liquid foods. Therefore, we designed two experiments, as follows: in Experiment 1, we employed a questionnaire to primarily measure participants’ eating intention (using a 9-point scale) for different foods (solid food and liquid food) through the priming of negative emotions. We hypothesized that individuals with negative emotions were likely to have a higher eating intention for solid food (highly chewable food) than liquid food (less chewable food). In Experiment 2, we screened individuals with high and low emotional eating habits using a questionnaire to further identify the effect of emotional eating on the intention to eat different foods (solid food/liquid food and highly chewable food/less chewable food) while experiencing negative emotions.

## 2. Experiment 1

### 2.1. Methods

#### 2.1.1. Study Design

The experiment adopted a within-subject design (food style: solid vs. liquid), with the rating scores of eating intention as the dependent variables.

#### 2.1.2. Participants

We determined the required sample size for Experiment 1 by G*Power 3.1 and then estimated the effect size to be small (η^2^ = 0.05). On the basis of an α value of 0.05 (two-tailed) and a power of 0.90, we found the required minimum number of participants for Experiment 1 to be 70. Finally, a total of 147 participants (female = 118) aged between 18 to 30 (*M* = 20.29, *SD* = 3.25) years were recruited for Experiment 1 through online recruitment information, meeting the minimum sample size (70). The study protocol was approved by the Ethics Committee of South China Normal University.

#### 2.1.3. Stimulus

A total of 5 types of foods, namely purple potato, maize, black soya bean, mango, and soybean, each in two forms—solid and liquid—were evaluated in this study. A total of 10 color photographs of the foods with a white background were chosen from the public archive, available online at http://baidu.com/ (accessed on 22 May 2021), and then standardized using Photoshop CS6 (Adobe Systems Software Ireland Ltd., Dublin, Ireland). All photographs were 500 × 300 pixels.

**Figure 1:**
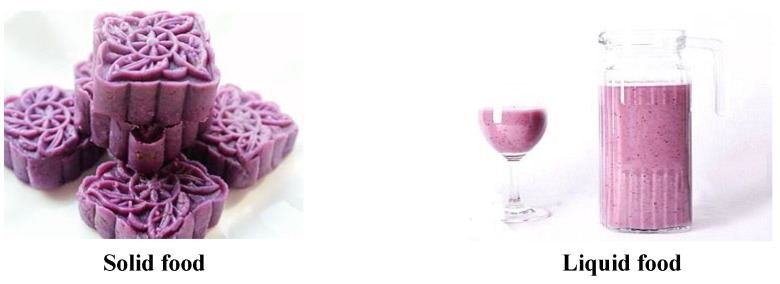
Profiles of different foods.

**Figure A1:**
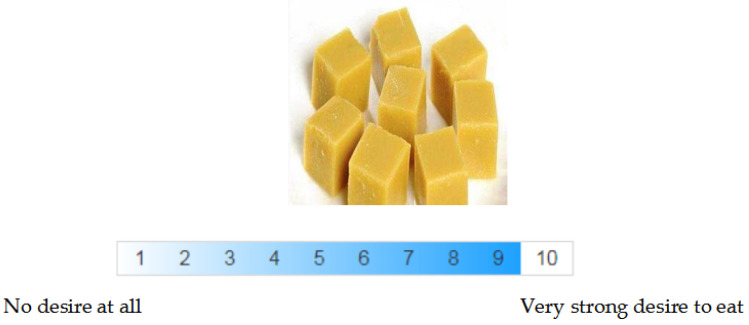
The picture of soy products (Solid food).

**Figure A2:**
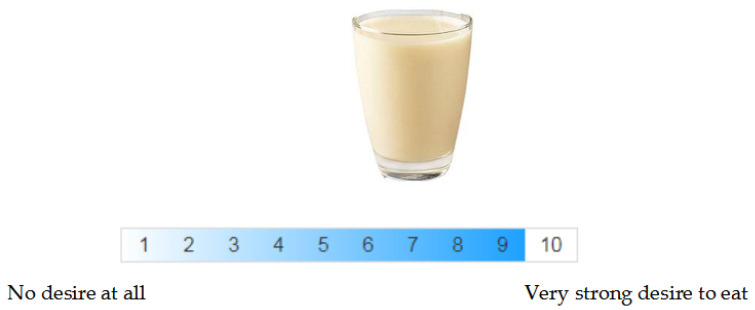
The picture of soy products (Liquid food).

**Figure A3:**
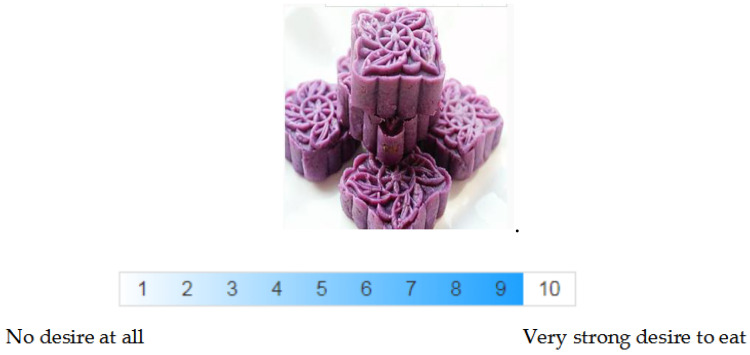
The picture of purple potato products (Solid food).

**Figure A4:**
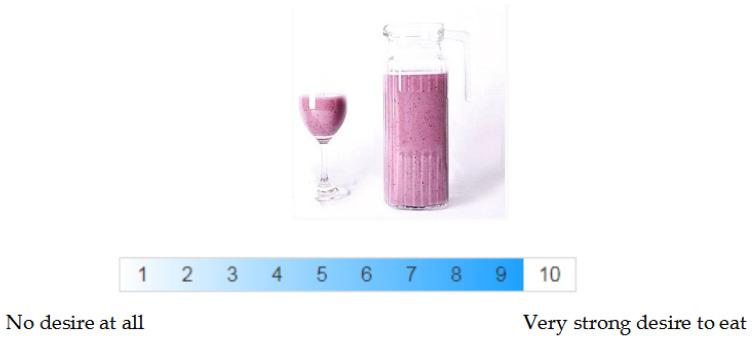
The picture of purple potato products (Liquid food).

**Figure A5:**
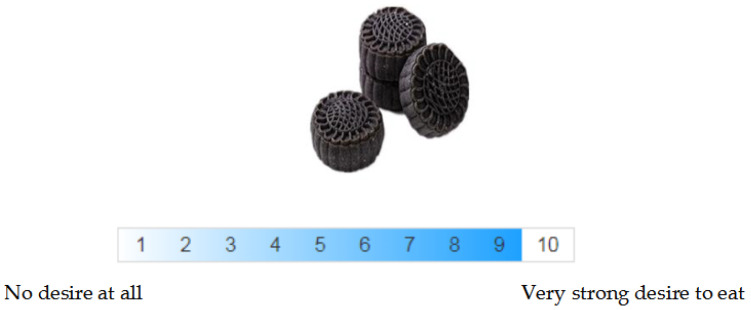
The picture of black bean products (Solid food).

**Figure A6:**
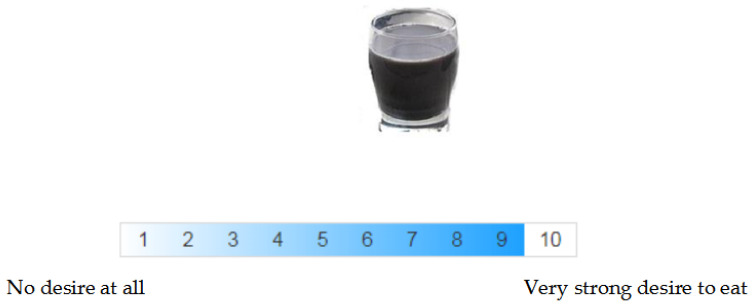
The picture of black bean products (Liquid food).

**Figure A7:**
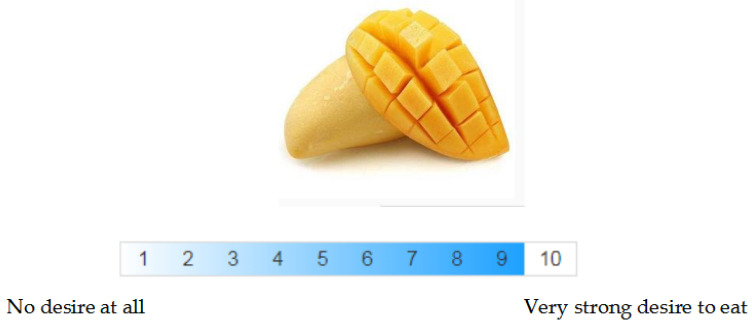
The picture of mango products (Solid food).

**Figure A8:**
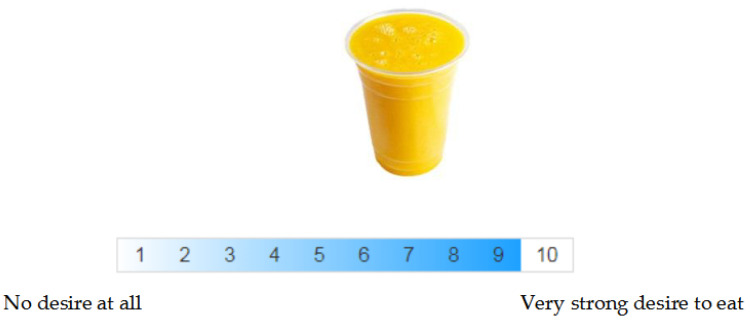
The picture of mango products (Liquid food).

**Figure A9:**
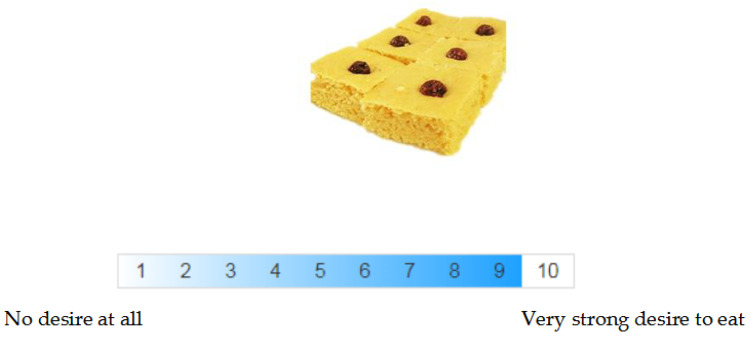
The picture of corn products (Solid food).

**Figure A10:**
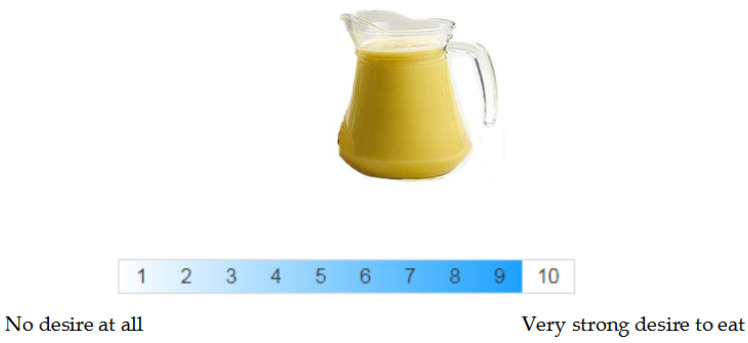
The picture of corn products (Liquid food).

Nineteen participants who had not previously participated in our experiment (10 female, *M* = 23.26, *SD* = 5.04) judged the familiarity of each food on a 7-point scale, with ‘1’ and ‘7’ denoting low and high familiarity, respectively. The chewiness of the food was also assessed on a 7-point scale, with ‘1’ and ‘7’ representing low and high chewiness degrees, respectively. The results of RM-ANOVA revealed no significant differences in the familiarity of the foods (see Table 1). However, we observed significant differences in chewiness degrees between solid and liquid foods. The mean scores of different foods regarding chewiness degree and familiarity are summarized in Table 1 (see Appendix A Figure A1, Figure A2, Figure A3, Figure A4, Figure A5, Figure A6, Figure A7, Figure A8, Figure A9 and Figure A10). Examples of the materials used in the present study are illustrated in Figure 1.

There were significant variations in individuals’ intentions to eat. If the food materials in this experiment were inherently different, the subjects’ intentions to eat the foods in the experiment differed under neutral conditions, a phenomenon that markedly interfered with the study’s purpose. Therefore, to better investigate the effects of individuals’ eating intentions toward the two food types while experiencing negative emotions, we randomly selected a few subjects and then conducted a preliminary assessment of their eating intentions (under neutral emotional conditions) prior to commencing the formal experiment.

**Figure 2:**
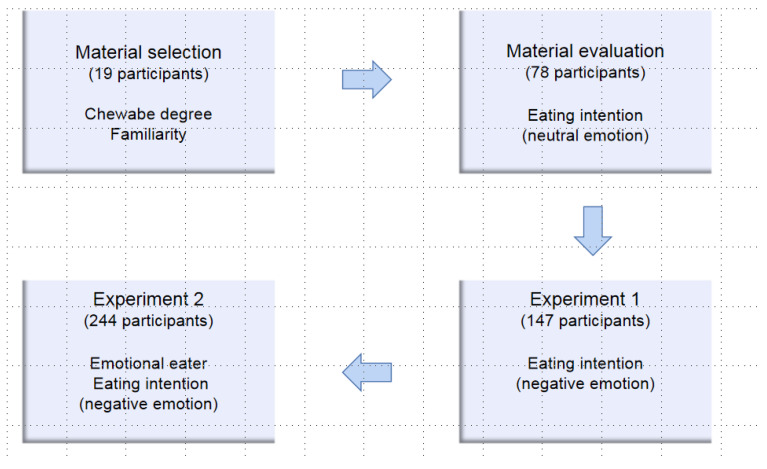
Diagrammatic representation of each experiment.

Next, we recruited a total of 78 Chinese college participants who did not participate in previous experiments aged between 18 and 30 (48 female, *M* = 20.53, *SD* = 2.14) years, then assessed their eating intention to eat different foods under neutral emotional conditions. This recruitment was conducted through an online program (see Figure 2). Participants were assigned to neutral emotional conditions according to the introductions. First, the participants under neutral emotional conditions were instructed to “calm your emotions, make yourself calm”. Five minutes later, they were presented with the second induction, after which their experience was rated on a 7-point scale (where 1 and 7 denoted very negative and very positive experiences, respectively). Next, the participants were sequentially shown 10 photographs of foods. They rated the photographs prior to being shown each food group and were then given an introduction: “Next, food production of ** (for example, *Ipomoea batatas*, See Figure 1) with the same energy and calories (the same food) will be presented, and you need to rate your eating intention, ‘How much do you wish to eat the food right now?’ for each food on a 10-point scale (a line scale). ‘1’ indicates a complete lack of desire to eat the food and ‘10’ indicates maximum desire to eat the food”. (See Appendix A Figure A1, Figure A2, Figure A3, Figure A4, Figure A5, Figure A6, Figure A7, Figure A8, Figure A9 and Figure A10).

Finally, we obtained 78 datasets with neutral initiation. After emotion induction, participants in the neutral condition group recorded a rating of *M* = 4.13 ± 0.87. Notably, their eating intention was not significantly different between solid and liquid foods (*p* > 0.05, See Table 1). These results demonstrated that the foods were suitable, and the participants did not have different eating intentions under neutral initiation.

#### 2.1.4. Procedure

**Figure 3:**
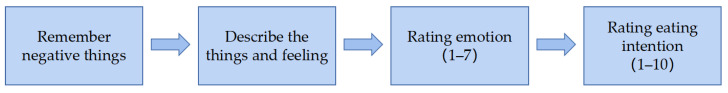
Procedure diagram.

Participants were first assigned to a negative emotional condition group according to the introductions, then instructed to ‘Please remember the things making you feel negative, and describe the things using some words and your feelings’. Five minutes later, they were required to rate their emotions on a 7-point scale (where 1 and 7 denote very negative and very positive responses, respectively). Next, the participants sequentially were shown 10 photographs of foods. Before rating the photographs in each food group, they were given the following introduction: “Next, food production of ** with the same energy and calories (the same food) will be presented, and you need to rate your eating intention, ‘How much do you wish to eat the food right now?’ for each food on a 10-point scale (a line scale). ‘1’ indicates a complete lack of desire to eat the food and ‘10’ indicates maximum desire to eat the food”. (See Appendix A Figure A1, Figure A2, Figure A3, Figure A4, Figure A5, Figure A6, Figure A7, Figure A8, Figure A9 and Figure A10) (See Figure 3).

### 2.2. Results and Discussion

#### Manipulation Check

**Figure 4:**
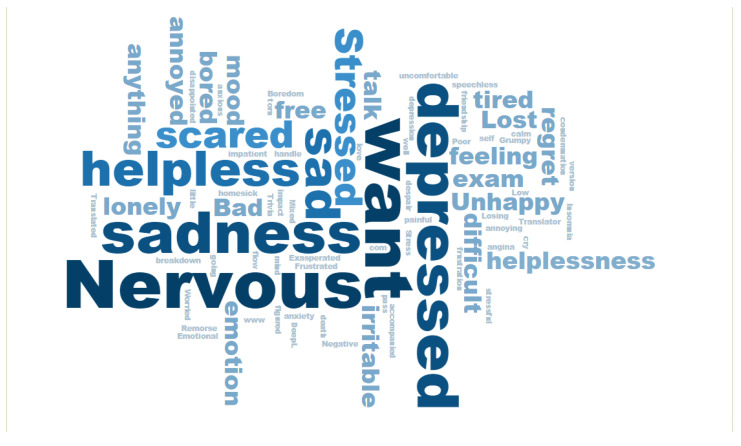
Profiles of negative emotions among participants.

The final rating score of the emotions under negative conditions was *M* ± *SD* = 3.85 ± 1.15. Moreover, our experiment triggered negative feelings among individuals, as evidenced by their subjective reports of emotions (see Figure 4). We used the analysis of word clouds to describe the participants’ emotions [49]. The size of a word represents how often subjects used it when they described their emotional state through verbal language. The more frequently a word appears, the larger its size.

The results showed that the main effect of food style was significant in eating intention [*t* (114) = 2.86, *p* *=* 0.001, Cohen’s *d* = 0.23, 95%CI (0.11, 0.58)] under negative emotion conditions. Notably, participants preferred solid food (5.83 ± 1.79) over liquid food (5.48 ± 1.82).

The results from Experiment 1 further demonstrated that participants reported a higher eating intention toward solid foods than liquid food under negative emotion conditions, in line with our hypothesis. Notably, food intake was influenced by the participant’s traits, such as emotional eating, which was defined as negative emotions while eating. Therefore, we hypothesized that emotional eating mediated the eating intention toward solid food (highly chewable food) in the context of negative emotions.

## 3. Experiment 2

### 3.1. Method

#### 3.1.1. Experimental Design

This experiment adopted a single-factor, between-subject design (emotional eating: high vs. low), with rating scores of food perception and eating preference as dependent variables.

#### 3.1.2. Participants

A total of 244 participants aged between 18 and 30 (38 male, average 19.39 ± 0.65) years were enrolled in Experiment 2. Participants were requested to complete a Dutch Eating Behavior Questionnaire (DEBQ, 13 items), and items were rated in terms of their frequency on a 5-level scale (1–never, 2–occasionally, 3–sometimes, 4–often, and 5–always). For example, ‘When you are angry, do you have the desire to eat?’ (emotional eating, α = 0.923) [50,51]. This study used a revised Chinese version of the DEBQ, and researchers found that the scale had good reliability (0.76–0.94) in the Chinese population (x2/d-2.38, CFI = 0.90, TLI = 0.89, RMSEA = 0.05, SRMR = 0.06) [52]. We stratified the participants into high and low emotional eating groups using the method of 27% based on participants’ scores of emotional eating. The pre- and post-percentage 27% grouping method is one of the more common methods of classifying high and low groups in psychology [53], so in this study, for the classification of high and low affectivity, we chose the before and after 27% grouping method. Finally, 85 (*M* ≥ 38) and 64 (*M* ≤ 23) participants were categorized into the high and low emotional eating groups, respectively. The study protocol was approved by the Ethics Committee of South China Normal University.

#### 3.1.3. Stimulus

The stimulus of Experimental 2 was similar to that used in Experiment 1.

#### 3.1.4. Procedure

Experimental 2 was conducted in a similar manner to Experiment 1 (See Figure 3).

### 3.2. Results and Discussion

#### 3.2.1. Manipulation Check

The final rating score of emotions under the condition was 3.93 ± 1.04 after emotion induction. The results revealed that food style had a significant effect on eating intention: *F* (1, 158) = 15.90, *p* *<* 0.001, η^2^ = 0.091. Notably, participants preferred solid foods to liquid foods. Moreover, emotions had a significant effect on eating intention: *F* (1, 158) = 11.44, *p* *=* 0.001, η^2^ = 0.068. The interaction effect of emotional eating and food style was not significant in eating intention: *F* (1, 158) = 1.52, *p* *=* 0.22. The mean rating scores for different foods of participants under negative emotion conditions are summarized in Table 2.

#### 3.2.2. The Relationship between Emotional Eating and Food Preference under Negative Emotion Conditions

Pearson’s correlation coefficients revealed a positive correlation between the eating intention toward solid food (r = 0.22, *p* < 0.01) and liquid food (r = 0.24, *p* < 0.01) with emotional eating scores.

**Figure 5:**
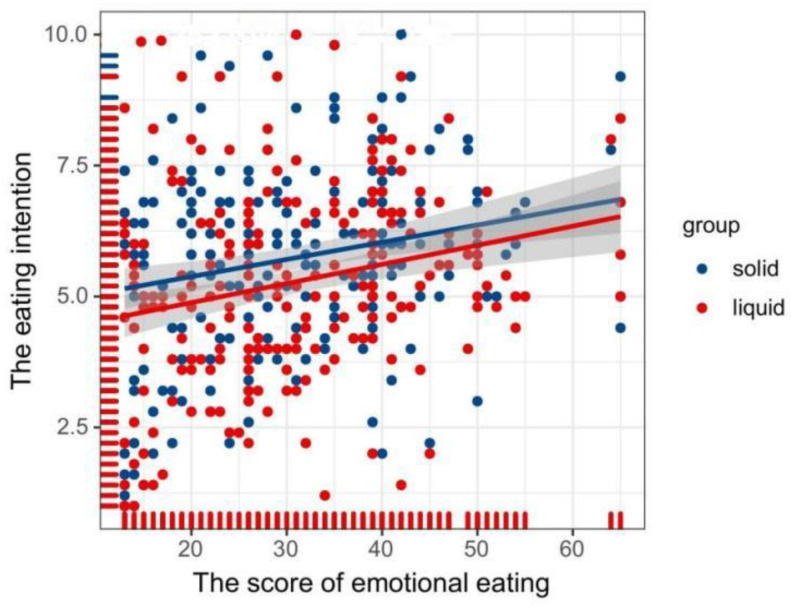
Line regression plot showing the relationship between eating intention with emotional eating scores. Note: Each point represents each participant in the target dataset.

Next, we conducted a linear regression analysis to further investigate the relationship between scores of emotional eating with solid and liquid foods. The results showed that the emotional eating score was a significant predictor of eating preference for solid food (R^2^ = 0.050, *β* = 0.22, *p* < 0.001) and liquid food (R^2^ = 0.057, *β* = 0.24, *p* < 0.001) (see Figure 5).

The results from the PROCESS analysis revealed that negative emotions and the intention to eat solid food were significantly influenced by emotional eating (*p* < 0.05), which has a negative moderating effect. The contribution of its moderating effect to the variance was close to 1.5%.

The results of Experiment 2 indicated that participants with high levels of emotional eating preferred solid foods under negative emotion conditions compared with their counterparts with low levels of emotional eating. Overall, these results demonstrate that emotions significantly affect preferences for highly chewable foods, which was in line with our hypothesis.

## 4. Discussion

The results from the present study showed that the intention toward different foods to eat is markedly influenced by emotions. Participants preferred to eat solid foods over liquid foods under negative emotion conditions when the foods had the same calories and sugar, which demonstrated a preference for highly chewable foods. Our results were consistent with findings from previous studies, which have demonstrated that emotions influence people’s eating behaviors [4,13,31,54]. Our results affirmed the effect of emotions on the food type we chose, consistent with findings from previous studies that have shown that people tend to prefer high-sugar, high-calorie, and other types of food when they have a negative mood [55,56].

The results from Experiment 2 revealed that emotional eating was significantly correlated with the intention to eat solid foods. The emotional eating score was a significant positive predictor of the intention to eat solid food. Moreover, we found significant differences in the intention to eat solid foods between individuals with high and low levels of emotional eating, which proves a preference for highly chewable foods once again. The results were in line with our hypothesis and consistent with previous findings that emotional eaters have higher eating intentions toward food [57,58]. Chewing, an essential behavior in daily life that is closely associated with hedonistic (emotional) systems in the brain, has been shown to relieve negative emotions [59]. For example, a previous study found that chewing behavior was associated with neurotransmitters; a reduction in chewing behavior could, to some extent, activate monoamine transmitters in the hippocampus and increase negative emotions. Notably, the inhibition of neurotransmitters in critical areas increases anxiety [39]. Previous studies have shown that monoamine neurotransmitters play a role in many physiological activities, such as emotion, arousal, and reward [60], while corticosterone has also been shown to be an indicator of the stress response. Results from previous research have demonstrated that mice with reduced chewing stimuli exhibited higher levels of corticosterone [40,41]. The results of the present study also showed that the chewiness of solid food was higher than that of liquid food, suggesting that solid food has a higher chewiness, which may help individuals relieve their negative emotions. Therefore, individuals have a high eating intention for solid food under negative emotion conditions. Emotional eating, which refers to overeating or binge eating in response to emotions, has been linked to the regulation of these undesired emotions [61,62]. In other words, emotional eaters are more likely to use eating behavior as a form of emotional regulation. On the basis of our results, it is evident that individuals with high levels of emotional eating prefer solid foods more than their counterparts with low levels of emotional eating. Therefore, we concluded that individuals under negative emotion conditions have a higher preference for solid food. We also proposed that the high preference for highly chewable foods under negative emotion conditions could be due to the effect of chewing on emotion relief.

## 5. Future Research and Limitations

This study had some limitations. Firstly, we only hypothesized that solid foods have better chewing properties, which can help individuals to alleviate emotions and ultimately cause them to have a higher preference for solid foods. Secondly, we did not consider information on subjects’ BMIs. Although BMI may affect the intention to eat, it was not well controlled for in this study; thus, it may have affected the results. However, we sampled university students from southern China, a region that ranks low in terms of BMI nationally according to the CDC—BMI values are generally relatively low, thus ensuring some control for the effect of BMI in the experiment. Thirdly, we did not realistically measure the effect of solid and liquid foods on alleviating negative emotions. Future studies are expected to explore this issue. Since obese individuals have a higher level of emotional eating than their healthy counterparts [58], they are more likely to adopt eating behaviors to relieve emotional stress; thus individuals, who reduce their emotional eating are more likely to successfully lose weight. In addition, during the arousal of negative emotions, we mainly initiated the sadness emotion in order to continue exploring an individual’s intention to eat under other types of negative emotions. Since the preference for food in this study was mainly based on one kind of picture, it may be difficult for participants to establish a relationship between solid food and emotion. Therefore, future explorations are expected to target individuals’ preferences for solid foods under negative emotion conditions by analyzing real behaviors across different chewable foods. Further studies are also needed to elucidate chewing preferences among individuals.

## 6. Conclusions

In summary, our findings revealed individuals’ preferences to eat solid food under negative emotion conditions. Notably, it is evident that individuals with negative emotions may prefer chewy foods, which provides new insights into the relationship between foods and negative emotions. Collectively, these results provide a basis for future food marketing and the design of new foods.

## Figures and Tables

**Table 1:** Mean scores of different foods on chewiness degree and familiarity.

	Solid	Liquid	*t*	*p*	Cohen’s *d*	95%CI	
	M ± SD	M ± SD				Low	Up
The chewable degree	4.48 ± 1.65	2.89 ± 2.53	−1.86	0.007	1.46	0.49	2.69
The familiarity	4.40 ± 1.60	4.90 ± 1.23	6.70	0.095		−1.11	0.10
Eating intention	5.89 ± 1.79	5.82 ± 1.98	0.41	0.68		−2.26	0.39

Note: *t*, the *t*-value is a test statistic; *p*, the *p*-value is the probability of the sample observation or more extreme outcome occurring when the original hypothesis is true. The smaller the *p*-value (less than 0.05), the more significant the difference between the two groups. Cohen’s d is the magnitude of the effect size, which is the number of universities that can determine whether a study with a significant difference has real significance or importance. 95%CI, confidence interval.

**Table 2:** Mean scores of eating intention toward solid and liquid foods among patients experiencing negative emotions.

	Low Emotional Eating (M ± SD)	High Emotional Eating (M ± SD)	*F*	*p*	η^2^
Solid food	5.36 ± 2.06	6.13 ± 1.54	7.23	0.008	0.04
Liquid food	4.81 ± 2.16	5.84 ± 1.46	12.83	<0.001	0.08

Note: *F* is the ratio of the two means (effect term/error term). The larger the *F* value, the more significant the effect between treatments. *p*, the *p*-value is the probability of the sample observation or more extreme outcome occurring when the original hypothesis is true. The smaller the *p*-value (less than 0.05), the more significant the difference between the two groups. η^2^ is the magnitude of the effect size, which is the number of universities that can determine whether a study with a significant difference has real significance or importance.

## Appendix A

You are about to be presented with a range of soy products, each with close to the same number of calories, and you are invited to rate the food on a 10-point scale of preference based on your current reality, with higher values indicating that you want to eat the food more.

You are about to be presented with a range of purple potato products, each with close to the same number of calories, and you are invited to rate the food on a 10-point scale of preference based on your current reality, with higher values indicating that you want to eat the food more.

You are about to be presented with a range of black bean products, each with close to the same number of calories, and you are invited to rate the food on a 10-point scale of preference based on your current reality, with higher values indicating that you want to eat the food more.

You are about to be presented with a range of mango products, each with close to the same calorie count, and you are invited to rate the food on a 10-point scale of preference based on your current reality, with higher values indicating that you want to eat the food more.

You are about to be presented with a range of corn products, each with close to the same number of calories, and you are invited to rate the food on a 10-point scale of preference based on your current reality, with higher values indicating that you want to eat the food more.
